# Patient participation in self-monitoring regarding healthcare of heart failure: an integrated systematic review

**DOI:** 10.1186/s12875-025-02757-6

**Published:** 2025-03-01

**Authors:** Sophia Olofsson, Hanna Josephsson, Maria Lundvall, Peter Lundgren, Birgitta Wireklint Sundström

**Affiliations:** 1 Faculty of Caring Science, Work Life and Social Welfare, University of BoråS, Allégatan 1, Borås, 501 90 Sweden; 2 Department of Anesthesiology, Operation and Intensive Care, Region Västra Götaland, Sahlgrenska University Hospital, Gothenburg, Sweden; 3 Primary Health Care Centre Valdemarsvik, Region of Östergötland, Valdemarsvik, Sweden; 4 University of Borås, Allégatan 1, Borås, 501 90 Sweden; 5 Department of Molecular and Clinical Medicine, Institute of Medicine, Sahlgrenska Academy, University of Gothenburg, Gothenburg, Sweden; 6 Department of Cardiology, Region Västra Götaland, Sahlgrenska University Hospital, Gothenburg, Sweden

**Keywords:** Heart Failure, Primary care, Self-care monitoring, Patient participation, Digitalisation, Integrated systematic review

## Abstract

**Background:**

Self-monitoring in cases of heart failure (HF) can lead to improved health and early detection of states of illness, potentially avoiding unnecessary hospitalisation. Legislation emphasizes the importance of patient participation in health care. This is possible and simplified due to the ongoing digitalisation within the healthcare system. The aim of this study was therefore to describe existing research knowledge on patient participation in self-monitoring regarding healthcare of HF, in the context of digitalisation of healthcare.

**Methods:**

A systematic literature review with an integrative approach was conducted February 2021 (6 years) and April 2024 (9 years). The review consisted of 12 articles accumulated from four databases. The review was performed in line with the standards of the PRISMA statement, registration number: PROSPERO 2021:244,252.

**Results:**

A total of twelve studies were included, both quantitative and qualitative research. The studies had a wide international spread and included a total of *n* = 1393 patients aged between 52–77 years, predominantly men. Various aspects of patient participation are the three themes: ‘Self-care ability*’*, ‘Interaction with healthcare professionals*’*, and ‘*Empowerment and Individual preferences’.* The results indicate that self-monitoring has a predominantly positive effect on self-care behavior and satisfaction with care. Increased awareness and confidence in patients´ own self-care abilities were reported especially in qualitative studies. Through the use of self-monitoring, information and knowledge about HF led to increased control of the disease. Additionally, differences between qualitative and quantitative studies are demonstrated even in this partial result. The qualitative studies showed an increased understanding of disease situations, but corresponding conformity is not shown in quantitative research, and an increased level of knowledge is not yet proven.

**Conclusions:**

The fact that there is a lack of empirical data in this field of research and that the available data is not coherent indicates that additional studies are required. In step with increased digitalisation and that great responsibility is placed on patient participation, there is a demand for patient studies that embrace a pronounced patient perspective with individual components of self-monitoring.

**Supplementary Information:**

The online version contains supplementary material available at 10.1186/s12875-025-02757-6.

## Background

Heart failure (HF) is a clinical syndrome associated with high morbidity and mortality. Worldwide, more than 26 million people are diagnosed with HF, leading it to be described as a ‘global pandemic’ [1]. In Western countries, it is one of the most common causes for recurrent hospitalisation, not only causing significant suffering for patients but also placing a heavy burden on the healthcare system. Patients with HF often present typical symptoms e.g. fatigue, breathlessness, ankle swelling, and signs of e.g. pulmonary oedema and peripheral oedema. These symptoms occur due to reduced cardiac output and/or elevated intracardiac pressures at rest or during stress, due to a structural and/or functional cardiac abnormality [2]. Patients with HF are dependent on long care contacts with different caregivers [3] and usually care takes place in several different places, for example in hospitals, hospital-affiliated HF-wards, and in primary care. Primary care has a central role in the care of patients with HF [4] with the goal to promote the patient´s belief in their own abilities in self-care [5, 6] and supporting self-care confidence [7]. To optimize self-care ability, patients must understand the causes and connections of their symptoms [3]. Educational aids can support this by providing tailored health information. In some cases, mobile devices are applied. The World Health Organization [8] defines mHealth as healthcare facilitated by wireless mobile devices, from simple SMS to complex phone applications. Self-care monitoring is a decision-making process where HF patients detect, interpret and respond to bodily changes that may indicate a deterioration in their condition at home [9, 10]. However, self-care monitoring with different tools such as information and communication technology, medical devices or paper-based material can be a challenge for patients with HF and varying experiences [9]. There are some patients that perceive mHealth tools to be supportive for identifying changes in HF symptoms [9].

Due to the increasing burden associated with HF and other chronic medical conditions, digital technologies continue to play an increasing role in transforming healthcare delivery [11, 12]. In this ongoing development of care and in relation to European guidelines (from 2016; 2021), the use of self-monitoring and telemonitoring has increased, with monitoring of signs and symptoms of HF as an integral part of self-care [3, 13]. Self-monitoring in HF can decrease the number of days of care spent in hospitals [14–16] and increase the likelihood of survival [17]. According to the Swedish Patient Act [18], healthcare must as far as possible be carried out in consultation with the patient. The Health and Medical Services Act [19] stresses the importance of healthcare being based on respect for patient participation, patients´ self-determination, and integrity. However, adopting a patient perspective on new technology presents challenges [20, 21]. Patient self-monitoring is a relatively new approach for primary care that requires evaluation from both caregivers and patients. Other challenges for patients with chronic diseases, where self-monitoring can be seen as a tool to increase their role in self-care and treatment, depend on patients´ willingness to use modern technology [22] and to be actively involved in their own care [23].

Participation needs to be based on the individual´s expectations and needs. Patients´ preferences and experiences are both important parts of describing patient participation. By first identifying what is important to the individual, a common understanding can be reached, allowing the opportunity to support patients’ experiences of patient participation [23]. Needs and wishes may vary. Some patients do not want to take full responsibility for decisions. In such cases, increased responsibility could lead to perceived limited patient participation [24].

Similarly, the importance of mutual trust with the establishment, between caregiver and patient, is emphasised through open communication with room for dialogue [25], influenced by shared information, and knowledge about their own care [26]. Shared decision-making is characterised by a person-centred participation in an ongoing dialogue process between the caregiver and the patient [24]. Patients´ experience of being involved is further described in terms of feeling responsible for their own care, discussing medication and treatment [25], and applying knowledge about symptoms, illness, and treatment [27].

There are challenges in self-care management for complex medical conditions, e.g. regarding patients´ difficulties in remembering tasks that need to be done [28]. Nevertheless, it is reported that patients see opportunities in self-monitoring, believing it to be helpful and supportive. However, they also wished that caregivers had access to their health data and were aware of their condition. Positive attitudes toward using self-monitoring as a tool in coping with disease are shown, but at the same time, patients want access to caregiver support [9]. Healthcare providers describe the use of self-monitoring in HF as an effective method that enables a detailed picture of the patient's condition, which can increase patient participation [29, 30]. Human encounters are highly valued [28, 9, 31–33]. In addition, the perceived usefulness is significantly associated with the intention to use self-monitoring when it comes to older patients with HF [34].

Furthermore, reports have focused more on patients' satisfaction regarding digital monitoring in HF rather than their experience of participation. Thus, to adopt a genuine patient perspective, it is important to identify and clarify current knowledge on patient participation in self-monitoring of HF, to be able to meet the growing digitalisation of healthcare. Also, knowledge about patient experiences and expectations are of utmost importance to be able to design and implement digital solutions in a clinical patient-oriented setting. Active participation and having influence are deemed as participation [23]. Consequently, in this study patient participation is defined as the individual having influence over their own care and health. The aim of this study was therefore to describe existing research knowledge on patient participation in self-monitoring regarding healthcare of HF, in the context of digitalisation of healthcare.

## Methods

An integrative systematic review was undertaken using the PRISMA guidelines [35] and follows the methodology outlined in the PROSPERO registered protocol (Database registration number: CRD42021244252). The review method used included studies with diverse methodologies, with a process involving problem identification, literature search, data evaluation, data analysis and reporting [36]. Good ethical practice in preparing and publishing systematic reviews were applied, aiming for transparency, accuracy and avoidance of plagiarism [37] and adhered to ethical principles of the Declaration of Helsinki [38].

### Literature search strategy

The literature search was conducted in February 2021 in the databases PubMed, CINAHL and Web of Science. An updated and extended search was conducted in April 2024 in the databases PubMed, CINAHL, Web of Science and ProQuest Dissertations & Theses Index (PQ_DT). A specialist subject librarian assisted in the development of the search strategy and implemented the use of the PICO [39] as follows: P: Patients with HF I: Self-monitoring at home C: No self-monitoring O: Patient participation.

The search terms were based on the purpose, and research questions. European Society of Cardiology Guidelines from 2016 and 2021 [3, 13] became the guiding principle for the search period. The search terms and their synonyms were derived by using MeSH terms and "Cinahl Subject Headings", in addition to a manual search. Web of Science and PQ_DT use Author keywords and Keywords plus. The search terms were combined using Boolean operators. Test searches were first conducted to establish the correct combinations of search terms. The CINAHL search strategy is available in Additional file 1.

The inclusion criteria were (i) peer-reviewed studies, (ii) published between January 2015 and March 2021 (6 years); and between January 2015 and April 2024 (over 9 years) and (iii) written in English. Exclusion criteria were studies on (i) children (≤ 18 years), (ii) without ethical consideration, (iii) literature reviews, systematic literature reviews and pilot studies and studies (iv) with focus on technology.

Articles were divided between two authors (SO and HJ; updated review ML and BWS) and independently screened against the inclusion/exclusion criteria, first by titles and then by abstracts. All researchers had regular meetings to discuss the process and to strengthen selection reliability. These discussions continued until consensus was reached among all authors. Overall, 306 abstracts were reviewed, following removal of duplicates. This resulted in 240 remaining abstracts for screening; from these, abstracts were excluded as not relevant; 63 remained. From these, additional abstracts were excluded, and 37 articles for full-text assessment were selected for eligibility. In the updated review 54 new records were reviewed; one remained for full-text assessment, but was excluded as not relevant.

**Fig. 1:**
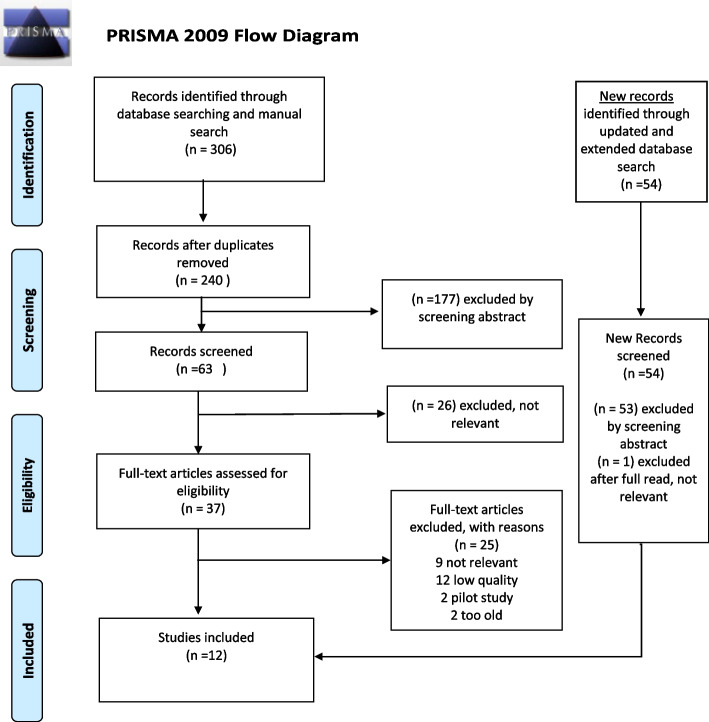
Inclusion- and exclusion process

The selected articles, deemed relevant by at least one of the authors, were evaluated with methodological and/or theoretical rigor on a 3-level scale (high, medium or low) [40, 41]. After the quality review and further discussions, nine articles were judged no longer relevant; two articles were considered too old; two were pilot studies; and twelve were considered of poor quality. A total of 12 articles were assessed as relevant to the study's purpose and of sufficient quality for final inclusion (Fig. 1). A summary of included studies is shown in Table 1.

**Table 1:** Summary of the reviewed studies (*n* = 12)

Authors First author (Year) Country	Aim and objectives	Data collection, key measurements	Major findings relevant to the review	Quality
Chantler et al. [53] Great Britain (UK)	To evaluate patients' experiences of self-monitoring with a focus on usability	Mixed methods Participants: 58	Increased: Relatedness with caregivers, sense of context, autonomy, control, awareness, responsibility, confidence, determination Need for: Individualised communication, more contact with care providers	High
Hägglund et al. [46] Sweden	To evaluate how self-monitoring influenced self-care behaviour, quality of life, hospital admissions and heart failure knowledge for heart failure patients	RCT Optilog Participants: 72 Control group: 40 Intervention group: 32	Self-care measured with EHFScB. Better after 3 months (p < 0.05) Knowledge measured with DHFK. No difference after 3 months (p = 0.4)	High
Hägglund et al. [55] Sweden	To investigate whether the situation-specific theory of heart failure can be applied in the context of heart failure patients using a self-monitoring service (Optilog) to support their self-care	Semi-structured interviews Optilog Participants:17 Recruited from Hägglund (2015)	Increased knowledge, insight and awareness of self-care, increased self-care ability in the form of improved symptom management, psychological support, a sense of not being alone, autonomy, confidence in one's own ability to make decisions, positive and negative opinions regarding advice from the decision support	High
Kiyarosta et al. [47] Iran	To evaluate the effect of smartphone application use on self-care behaviour in patients with heart failure	RCT My Smart Heart-app Participants: 120 Control group: 60 Intervention group: 60	Self-care measured with EHFScB Better after 3 months (p < 0.001)	High
Lycholip et al. [51] Netherlands	To assess the impact of self-monitoring on self-care behaviour, evaluating the dynamics of self-care, investigating contributing factors to changes in self-care and identifying a patient-adapted service that improves the patient's self-care	RCT TM + ICT-guided-DMS Participants: 118 Control group: 60 Intervention group: 58	Self-care was measured with the EHFScB No significant difference after 9 months (p = 0.77)	Medium
Melin et al. [48] Sweden	To evaluate whether self- monitoring improves self-care, as well as the impact on quality of life, disease knowledge and hospitalizations	RCT Optilog Number of participants: 72 Control group: 40 Intervention group: 32	Self-care was measured with the EHFScB Improvement after 6 months (p < 0.05) Knowledge was measured with the DHFK Häger knowledge after 6 months (p < 0.05)	High
Noel et al. [45] USA	To evaluate the effects of a self-monitoring service compared to usual care	RCT TTOC Participants: 74 Control group:43 Intervention group: 31	Significant improvement in enthusiasm and confidence that the self-monitoring was helpful for patients (p = 0.0001) No significant difference in how confident they are in their self-care (p = 0.914)	High
Somsiri et al. [44] Thailand	To investigate the effectiveness of a self-monitoring program on functional status, rehabilitation and satisfaction with care in Thai heart failure patients	RCT TTP Participants: 145 Control group: 73 Intervention group: 72	Satisfaction with care was measured with The Thai version of "the Satisfaction with Care Questionnaire" Significantly higher perceived satisfaction with their care after 6 weeks (p < 0.001)	High
Wagenaar et al. [49] Netherlands	To evaluate the impact of an interactive disease management platform on self-care	RCT e-Vita platform Participants: 300 Control group:150 Intervention group:150	The primary outcome was measures of self- care and secondary outcomes were health status, hospital stays and mortality. Self-care was measured with the EHFScB. Significant difference between the study groups regarding self-care after 3 months (p < 0.001) and 6 months (p = 0.070), but not after 12 months. No significant difference regarding knowledge	High
Ware et al. [54] Canada	1. To measure patient compliance 2. To explain sustained adherence based on participation, as well as examine patient characteristics and patients' perceptions of the self-monitoring service	Mixed methods Medley Participants: 231 Interviews n = 24	Quantitative result: Agreed with the statement that self- monitoring was important to them in managing their heart failure: 90.6% (after 6 months) 95.8% (after 12 months). Agreed that it would be useful for them to continue using self-monitoring. 87.4% (after 6 months) 93.9% (after 12 months) Qualitative result: Positive: Increased responsibility and guidance in self-care, improved relationship with care providers, increased control Negative: Experience that feedback did not mirror the whole and the context	Medium
Ware et al. [52] Canada	To evaluate the effect of self- monitoring, with regard to healthcare needs, clinical results, quality of life and self-care	Quantitative study Pretest- and posttest design Medley Participants: 156	Self-care was measured with the SCHFI divided into maintenance, management and confidence. There was a significant measured improvement in perceived self-care within maintenance (p > 0.001) and management (p = 0.01). No significant difference could be shown for the confidence score. No significant difference for self-confidence regarding self-care after 6 months (p = 0.23)	High
Yanicelli et al. [50] Argentina	To evaluate the effect of self-monitoring regarding improvement of self-care and adherence to treatment	RCT HTS Participants: 30 Control group:15 Intervention group:15	Self-care measured with EHFScB Better after 3 months (p = 0.004)	Medium

### Data analysis

Thematic analysis with a systematic and inductive approach was used [42, 43]. Familiarisation with the data involved several readings and re-readings of two authors (HJ and SO) to achieve a comprehensive understanding of the studies involved. By extracting meaningful units, which matched the purpose of the study and research questions, a `data-driven´ and open coding was applied. Identified codes were compared based on similarities and differences so that similar data were sorted into categories to identify patterns, relationships, and themes. Sorting into themes and sub-themes was done until the patterns were meaningful in relation to the purpose of the study. Since themes and sub-themes are abstractions of extracted data, these were controlled against the respective primary source, in order to verify the results. The interpretation was discussed and finally agreed upon by all the authors.

## Results

Nine out of 12 included studies used a quantitative approach and eight of these used a randomised controlled design [44–51]. One quantitative study used a pretest–posttest design [52]. In addition, there were two mixed method studies [53, 54] and one interview study [55]. The studies included were published between 2015 and 2020 and all internationally spread. Three studies were from Sweden, two from Canada and two from the Netherlands. The USA, Great Britain, Iran, Argentina and Thailand were each represented by one study. The total number of participants was *n* = 1393, with the ages between 52–77 years, predominantly male. The study settings varied and included acute and ambulatory settings, intensive care units, specialty heart function clinics and medical outpatient clinics for Chronic HF patients in hospitals. Study periods ranged from 3 months (90 days) to 15 months, with one study spanning nearly 3 years.

The findings constitute three themes: Self-care ability; Interaction with healthcare professionals; and Empowerment and individual preferences (Table 2). These themes are associated with a total of nine sub-themes, which are indicated in italics. Additionally, two overarching research questions are addressed.

**Table 2:** The results presented as themes and sub-themes

Themes	Sub-themes
Self-care ability	*Predominantly positive effect on self-care behaviour*
	*Increased insight and awareness*
	*Enhanced decision-making when seeking care*
Interaction with healthcare professionals	*A reach out to the caregiver*
	*Need for consulting a physician*
	*Need for individualised weekly counsel*
Empowerment and individual preferences	*Opportunities to gain knowledge*
	*Gaining control or being surveilled*
	*Predominantly positive effect on satisfaction with care*

### Self-care ability

This theme illustrates how patients perceive self-care ability influence, i.e. the ability to manage the disease in terms of self-monitoring. The studies used different telehealth programs, home telemonitoring system (HTS), applications for tele-monitoring (TM), and TM + information-and-communication-technology (ICT)-guided disease management system (ICT-guided DMS), e.g. Optilogg, My Smart Heart, e-Vita platform and the Medley program. The results vary, especially those regarding credence in self-care ability. This theme also includes three sub themes: *Predominantly positive effect on self-care behavior, Increased insight and awareness* and *Enhanced decision-making when seeking care.*

### Predominantly positive effects on self-care behavior

The quantitative studies, of which six [46–51], applied the European Heart Failure Self-Care Behavior Scale (EHFScB) in order to estimate self-assessment regarding self-care behavior. These results describe e.g. responses to symptoms including patients adjusting their diuretic dose and contacting a caregiver when needed. One study [52] used Self-Care Heart Failure Index (SCHFI), which also measures patients’ self-care behavior. Differences occur concerning to what extent self-care behavior has improved after respective intervention. On the other hand, results indicate significant positive effects on self-care behavior after interventions. Exceptions are for Lycholin et al. [51], where no improved self-care was measured, and the latest follow-up in Wagenaar et al. [49], where no improvement was shown in the long term. Continuous updating of e-health facilities was suggested to be helpful to sustain effects.

### Increased insight and awareness

Qualitative studies report that patients experience increased insight and awareness [54, 55] regarding the importance of self-care. The self-monitoring service is described as providing guidance in self-care by Ware et al. [54]. A similar result is found in Hägglund et al. [55], where daily reminders are emphasised.

### Enhanced decision-making when seeking care

Several studies regarding self-monitoring describe patients’ increased self-confidence and credence in self-monitoring [45, 53, 54]. In Ware et al. [54] feedback from caregivers are especially important for patients regarding whether to seek care or not. A similar result is found in Chantler et al. [53] in which “external medical audit” linked to self-monitoring provides a sense of inclusion. Leading to patients’ determination to manage the disease. Patients’ appreciation with support in decision-making is said to function as a transition from hospital care to self-care [55]. However, two quantitative studies show no significant difference regarding patients’ assertiveness after self-monitoring [45, 52].

### Interaction with healthcare professionals

This theme illustrates patients’ experience of interaction and dialogue with caregivers while using self-monitoring. Opportunities and possibilities for caregiver contact, beyond technical support, were created. However, there were some deficiencies, i.e. that the self-monitoring service did not depict the entirety of the state of health. Suggestions for improvement in the self-monitoring service are accounted for regarding information and notifications. The theme is described in three sub-themes: *A reach out to the caregiver, Need for consulting the physician* and *Need for individualised and weekly advice.*

### A reach out to the caregiver

Studies signify that the use of self-monitoring service has an impact on patient and caregiver relationships [53–55]. Patients who utilised self-monitoring services over an extended period expressed improved relationships with caregivers compared to ordinary care [54]. Similar results are found in Chantler et al. [53] where patients state that self-monitoring service created a connection with caregivers, providing a secure foundation for self-monitoring. Some of the patients in Hägglund et al. [55] expressed that the self-monitoring services provided support, security and conveyed a feeling of not being alone in their situation.

### Need for consulting a physician

Patients’ views concerning advice, feedback and injunctions via self-monitoring services are reported [53–55]. Patients who showed a lower degree of compliance claimed that exhortation and feedback did not fully capture the state of health context [54]. Some patients stated that the feedback did not always mirror their actual condition, and they eventually learned not to respond to certain warnings [54]. A similar response was found in Hägglund et al. [55]. Patients did not trust the advice via self-monitoring services and preferred consulting their physician before following digital recommendations.

### Need for individualised weekly counsel

It is stated that there is a need for individualised counseling and information about assessments mediated by caregivers on a weekly basis [53]. Patients described counseling via self-monitoring services as highly motivational. Contact options served not only as technical support but also ‘humanised’ the service by facilitating interaction and dialogue. Patients preferred messages instead of telephone calls [54]. They received automated telephone calls if they failed to carry out self-monitoring, which were percieved as irritating but at the same time as important reminder.

### Empowerment and individual preferences

The theme illustrates how patients, through self-monitoring, not only gain knowledge about HF but also experience an increased level of control and responsibility. The self-monitoring service is supportive when it comes to manage the chronic disease. However, the partial result lacks consensus. Furthermore, anxiety concerning comprehending medicinal values and taking responsibility for their interpretation have been reported. This theme also includes three sub-themes: *Opportunities to gain knowledge*, *Gaining control or being surveilled* and *Predominantly positive effect on satisfaction with care.*

### Opportunities to gain knowledge

Three of the quantitative studies included in this review compared HF knowledge differences based on Dutch Heart Failure Knowledge Scale (DHFK) [46, 48, 49]. HF knowledge increased after the intervention in two studies [48, 50], no differences between intervention group and control group were reported in one of these [46]. Patients reported no gained HF knowledge post intervention in the third study [49].

Qualitative studies report a more cohesive depiction concerning patients’ knowledge on HF. The use of self-monitoring service was seen as a possibility to gain knowledge [54]. Prominent aspects included weight, diet, physical activity, smoking cessation, and reduced alcohol consumption. Furthermore, increased knowledge about symptom changes was reported [53, 55]. Patients improved their ability to recognize and interpret symptoms through the self-monitoring service due to the fact that these included graphical evaluation diagrams. Experienced symptoms and objective data e.g. weight was interwoven to an entirety [55]. Patients also expressed increased awareness and knowledge how to act when symptoms occur [53].

Patients report a higher level of autonomy i.e. regulating medication and the ability to decide what measures required. Some advice from the self-monitoring service was neglected since patients claimed that they themselves took responsibility. They trusted their own decision-making ability, and the gained knowledge made them self-confident [55].

### Gaining control or being surveilled

Several studies describe control, autonomy, and responsibility as positive aspects [53–55]. Patients state that since it is challenging to manage a chronic disease, self-monitoring functioned as a tool to manage everyday life. They emphasised how the system encouraged independence, increased control and awareness about symptoms [53]. Similarly, a sense of control is reported [54]. As soon as medicinal values were uploaded, direct feedback via self-monitoring helped patients plan daily activities.

A sense of increased autonomy i.e. patients’ ability to regulate their own medication and by determining the necessary measures, is reported in one study. Some patients decided not to follow given advice since they deemed themselves responsible. This knowledge in turn led to increased self-assuredness [55].

Other aspects were highlighted in one study where patients experienced self-monitoring as intrusive, feeling a sense of surveillance that impinged on their privacy. For example, if caregivers noticed patients had been careless with their diet, patients sometimes avoided weighing themselves to prevent revealing deviant data. Concern about interpreting medicinal data is also reported [54]. Corresponding results showed increased awareness by patients, but they were still worried and unsettled to be responsible for interpretation of the data. Some claimed that they would rather assign interpretation to caregivers. Despite qualms concerning interpretation of data, patients in the above study also reported appeasement, increased self-esteem and goal fulfillment [53].

### Predominantly positive effect on satisfaction with care

Several studies report positive results regarding patients’ satisfaction, indicating that self-monitoring fulfilled needs related to symptom management [44, 45, 54]. Average values of care satisfaction were significantly higher than the control group after six weeks of self-monitoring [44]*.* Statistically significant improvements in enthusiasm and confidence regarding self-monitoring were reported [45]. Similarly, 30 days after discharge, patients state an evident increase in self-esteem compared to the control group.

### How is active participation described?

The result indicates that increased responsibility and insight into managing self-care interventions increases participation. It appears that self-monitoring serves as a tool for developing and applying necessary self-care arrangements. Increased awareness of the importance of self-care leads to active participation and enhanced decision-making compliance. Further, the result indicates increased responsibility and insight into healthcare interventions.

#### How is having influence over own care described?

The results indicate that self-monitoring could enhance relationships between patients and caregivers, thereby increasing influence over one's own healthcare. However, negative messages and advice were sometimes perceived as being neglected and excluded, leading to a lack of trust in the advice given. This lack of trust resulted in patients still require physician consultation.

## Discussion

One key finding of this review constitutes the shortage of studies that have specific focus on patient participation in self-monitoring regarding the treatment of HF. This was surprising given that telemedicine and self-monitoring in HF are identified as the `digital revolution´ and announced as a potential way of escalating HF multidisciplinary integrated care, but still at an early stage [56, 57]. In addition, patient participation in healthcare is highlighted by WHO [8] and in Swedish law [18, 19], although there is no consensus regarding the concept of patient participation [24, 26].

### Self-care ability

The qualitative result corroborates positive effects on self-care behaviour and depicts increased awareness and trust regarding self-care ability. Corresponding results are confirmed in patients with other chronic diseases, i.e. strengthened self-care by improved ability to manage illness and enhanced self-management capability after utilising self-monitoring [58, 59].

Earlier studies have also indicated varied results regarding patients’ active role in self-care. In addition, there are expectations on caregivers to take full responsibility for performing care. Older patients proved to be more negative towards self-care, whereas the younger wished for increased control [60].

### Interaction with healthcare professionals

Feedback on objective values such as weight, blood pressure, and symptom estimation do not necessarily fully capture disease states. Thus, this data emphasises the importance of influence in engaging in nurse-patient relationships and the need for patients to be treated as true individuals [61]. One earlier study shows that patient-centeredness is associated with empathy of the primary care provider [62].

### Empowerment and individual preferences

There is no consensus regarding patients’ empowerment and a possible connection between self-monitoring and knowledge related to HF. On the other hand, qualitative studies indicate that self-monitoring systems contributed to increased levels of control regarding symptoms versus treatment. This might promote patient participation. The results also show experiences of satisfaction with care in accordance with another study [63]. In addition, previous studies argue that patients’ satisfaction of care is closely linked to aspects of patient participation [64]. An important aspect of experiencing satisfaction with given care is to gain knowledge and explanations about one’s state of health. The more information obtained, the higher the level of satisfaction can be obtained.

However, deficient dialogue between patients and caregivers might dispute whether decisions are mutual or not [65]. Previous studies on patients with chronic medical conditions reported increased commitment and understanding of their health condition that provided a sense of empowerment to manage their own health [66–68]. The association between active patient engagement and beneficial effects on health outcomes are also shown [69]. One study informs and provides goals for the support of self-monitoring regarding patients with chronic HF [70]. Nevertheless, the result clarifies different needs concerning the responsibility for the interpretation of self-monitoring data and the possibility of `Asking for help´ in daily activity [71]. On the other hand, the decision to support was in some cases perceived as surveillance, thus encroaching integrity and autonomy. Previous research has demonstrated the importance of identifying patients’ preferences regarding participation in care to address basic individual needs [72, 73] and to establish a strong support system for outpatient care [70]. Previous research has also shown the challenges to describe the effects of self-management program on preferences for, and experiences of, patient participation in patients with long-term condition [74].

### Strengths and limitations

The broad aim and search strategy are limitations, but are also necessary to be able to capture the phenomenon of `patient participation´ in self-monitoring regarding healthcare of HF in the context of digitalization, despite the diversity in self-care interventions and self-care monitoring. The search period (post 2015) could be seen as too narrow and therefore also a limitation. However, this was a part of our strategy, to capture the latest research. We had to relate to the fact that the research area is relatively new. Furthermore and, for the same reason, the focus was on scientific studies with rigor results, therefore, pilot studies were excluded. All these factors that limited the search were deliberate choices to achieve the purpose of the study. Another limitation was that one study [45] of the 12 included articles studied self-monitoring in adult chronic diseases in general, not only for HF. However, after a deep study of this article we found that it could be accommodated to our purpose and question formulation, even though it could be seen as a concession since there is a lack of specific patient studies.

It is known that it can be complex and challenging to combine different research data [36, 75]. Complexity in the data presupposes a systematic and rigorous approach, especially in the analysis, to prevent bias. Many researchers strongly recommend building a team to carry out the literature review to ensure quality. Strengths in the present study refers to the search strategy. We utilised a systematic approach through the PICO model [39] twice, at three-year intervals. The first literature searches were conducted in three different databases and an updated and extended search in four databases. Furthermore, two of the authors conducted the first search strategy and two other authors conducted the second one, both times with regular support from a specialist subject librarian. To promote objectivity and a critical approach, the findings were additionally processed and discussed with all authors.

Despite the use of an experienced librarian in the search phase, the possibilities exist that the search methodology, inclusion- and exclusion-criteria did not capture all relevant studies. Another risk includes the possibility that publications may already have been missed in the first screening process, if the title did not include a patient perspective. An additional limitation is that we did not use The Cochrane Central Register of Controlled Trials (CENTRAL).

All analysed quantitative studies were carried out with repeated follow-ups and measurements over several months, which underpins good reliability. In combination with the fact that the results are based on previously well-known and validated instruments, a generalization becomes possible. The quality of a review is based on the included studies and therefore it is important that the studies are assessed and valued. Included articles were reviewed based on qualitative checklists. The articles that were judged to have average or high quality after the quality review were included in the study. The rest were excluded, for example, due to methodological flaws, too few participants, or ethical considerations not being clearly presented.

Something that strengthens the validity of this systematic review is that the patients in the included studies report similar results. The measuring instruments themselves are not dependent on the self-monitoring services.

## Conclusions

Even though there is a lack of accessible research on this topic, the results show that self-monitoring regarding health care of HF can facilitate patient participation in terms of improved self-care ability, increased experienced control, credence in ability and satisfaction with provided care. However, this result lacks concordant evidence. The fact that there is a lack of empirical data in this field of research and that the available data is not coherent indicates that additional studies are strongly required. This is especially important as great responsibility is placed on patient participation, patients’ own needs and preferences for participation in self-monitoring in HF and requires further acknowledgement and approval. In step with increased digitalisation there is a demand for patient studies that embrace a pronounced patient perspective with individual components of self-monitoring, for instance motivational elements and personal support from caregivers. Finally, the need to identify patients' individual preferences is emphasised to strengthen their participation in healthcare and improve health outcomes. Therefore, we recommend studies that deepen the knowledge in how different components influences patient participation.

## Supplementary Information

Supplementary Material 1.

## Data Availability

No administrative permissions were required for accessing databases as all were available to the authors.
