# Supplementary material for: Patient participation in self-monitoring regarding healthcare of heart failure: an integrated systematic review
Source: BMC Prim Care. 2025 Mar 1;26:60. doi: 10.1186/s12875-025-02757-6 (PMC11871632; doi:10.1186/s12875-025-02757-6)
Supplement: Supplementary file 1 — Supplementary Material 1. [file 12875_2025_2757_MOESM1_ESM.docx]

Additional file;

CINAHL Search Strategy Ran in February 2021 and April 2024

#1 “heart failure” OR “cardiac failure” OR “chf” OR “chronic heart failure” OR “congestive heart failure”

# 2 "self monitoring" OR “home monitoring" OR “Decision support” OR “Home health care information systems” OR "Decision Support Techniques" OR “home intervention system (HIS) “ OR “Decision support systems, management” OR “Telemonitoring” OR “home telemonitoring (HTM)” OR “telemedicine” OR “mHealth” OR “Mobile Health” OR “Health, Mobile” OR “Telehealth” OR “eHealth” OR “Telerehabilitation” OR “telenursing”

#3 “Participation, Patient” OR “Patient Involvement” OR “Involvement, Patient” OR “Patient Empowerment” OR “Empowerment, Patient” OR “Patient Participation Rates” OR “Participation Rate, Patient” OR “Participation Rates, Patient” OR “Patient Participation Rate” OR “Patient Activation” OR “Activation, Patient” OR “Patient Engagement” OR “Engagement, Patient” OR “consumer participation” OR “Care, Patient-Centered” OR “Patient Centered Care” OR “Nursing, Patient-Centered” OR “Nursing, Patient Centered” OR “Patient-Centered Nursing” OR “Patient Centered Nursing” OR“Patient-Focused Care” OR “Care, Patient-Focused” OR “Patient Focused Care” OR “Medical Home” OR “Home, Medical” OR “Homes, Medical” OR “Medical Homes” OR “Shared Decision Making” OR “Care, Self” OR “Self-Care” OR “Efficacy, Self” OR “Self efficacy” OR “patient education” OR “health literacy” OR “case management” OR “case manager” OR “self-care management” OR “empowerment”

# 4 #1 AND #2 AND #3
